# Experimental data of the aqueous NH_3_ and CO_2_ absorption at temperatures from 15 °C to 35 °C, NH_3_ concentrations from 5% to 15% and CO_2_ loadings from 0.2 to 0.6 measured with the Wetted Wall Column

**DOI:** 10.1016/j.dib.2018.02.047

**Published:** 2018-03-02

**Authors:** Stefano Lillia, Davide Bonalumi, Philip L. Fosbøl, Kaj Thomsen, Gianluca Valenti

**Affiliations:** a Politecnico di Milano, Dipartimento di Energia, Via R. Lambruschini 4, 20156 Milano, Italy; b Technical University of Danemark, Department of Chemical Engineering, Center for Energy Resource Engineering, Søltofs Plads, 2800 Kgs. Lyngby, Denmark

## Abstract

The absorption between aqueous NH_3_ and CO_2_ is studied using the Wetted Wall Column in order to show the effect of the solvent condition on the rate of reaction. A total of 27 different cases are investigated in the region defined by temperatures from 15 °C to 35 °C, NH_3_ concentrations from 5% to 15% and CO_2_ loadings from 0.2 to 0.6. The paper reports the data measured during the experiments, the experimental apparatus description and the experimental procedure. The data here presented are both the raw data measured with their uncertainty and the final value of the overall mass transfer coefficient. The overall mass transfer coefficient is the result of the raw data treatment explained in the research paper related to this data. The data here reported are analyzed in the paper by Lillia et al*.* (2018) [1].

## Nomenclature

**Acronyms**
VR: Value Read
FS: Full-Scale
WWC: Wetted Wall Column
Liq.: Liquid phase
Gas: Gas phase

**Symbols**
$K_{\mathrm{ov}}$: overall mass transfer coefficient for the CO_2_ transport [mol/(Pa m^2^ s)]
$P_{i}^{j}$: partial pressure of component i at position j [mol/s]

**Greek symbols**
$\varphi_{CO_{2}}$: CO_2_ flux absorbed by the solvent [mol/(m^2^ s)]

**Specifications Table**

**Table:** 

Subject area	*Environmental chemical engineering*
More specific subject area	*Absorption solvent characterization and rate of the absorption measurements*
Type of data	*Table of data and text*
How data was acquired	*The data are acquired by the Wetted Wall Column apparatus.*
	*The partial pressure of the CO*_*2*_*inside the reactor is measured considering the total pressure in the reactor with a pressure transducer (Rosmount 2088) and the CO*_*2*_*concentration with the CO*_*2*_*probe (VAISALA GMT 220).*
	*The CO*_*2*_*flow absorbed by the solvent in the reactor is measured considering the difference of the CO*_*2*_*concentration with the CO*_*2*_*probe (VAISALA GMT 220) before and after the reactor and the gas mole flow blown to the reactor.*
Data format	*Raw measurements with uncertainties and their analysis (the overall mass transfer coefficient).*
Experimental factors	*The solvent is prepared gravimetrically in terms of ammonia composition and CO*_*2*_*loading (defined as the rapport of CO*_*2*_*and NH*_*3*_*in the solvent).*
Experimental features	*A gas mixture of N*_*2*_*and CO*_*2*_*with a known composition flows in the Wetted Wall Column in contact with solvent with a known composition. The partial pressure of the CO*_*2*_*inside the reaction chamber and the CO*_*2*_*flux absorbed by the solvent are measured during the experiment for different compositions of gas and solvent. The result is the overall mass transfer coefficient which describes the dependence of the rate of absorption and the partial pressure of the CO*_*2*_.
Data source location	*Department of Energy of Politecnico di Milano, Milan (Italy)*
Data accessibility	*Data are provided within the attached excel file and within the article*
*Related research article*	*Experimental study of the aqueous CO*_*2*_*-NH*_*3*_*rate of reaction for temperatures from 15* *°C to 35* *°C, NH*_*3*_*concentrations from 5% to 15% and CO*_*2*_*loadings from 0.2 to 0.6*[1]

**Value of the data**

• Enhance the number of analyzed cases for the CO_2_ capture with ammonia solvents
• Raw data with their uncertainties provide information about the accuracy of the measures and the procedure used.
• Raw data are treated to compute the overall mass transfer coefficient under the hypothesis explained by Lillia et al. [1]. Other researcher can model the phenomenon under other hypothesis and treat the raw data with different procedures and compare the *K_ov_* results found.
• *K_ov_* value can be compared with other data in literature and new data measured in future works with the same method.

### 1 Data

The data provided by the paper are the Wetted Wall Column measurements for every case analyzed with the absolute uncertainty. The aim of the measurements is the calculation of the CO_2_ flux absorbed by the solvent and the partial pressure of the CO_2_ inside the Wetted Wall Column reactor. The data presented are the raw data measured by the Wetted Wall Column apparatus and their analysis following the procedure explained in the relative research article by [1]. The raw data treatment returns the overall mass transfer coefficient $K_{\mathrm{ov}}$ [mol/(m^2^ s Pa)] that correlates the difference of CO_2_ partial pressure in the reaction chamber and the CO_2_ flux absorbed by the solvent as defined in Eq. (1)

(1) $$\varphi_{{\mathrm{CO}}_{2}}=K_{\mathrm{ov}}\left(P_{{\mathrm{CO}}_{2}}^{\mathrm{gas}}-P_{{\mathrm{CO}}_{2}}^{\mathrm{liq}}\right)$$

where $\varphi_{{\mathrm{CO}}_{2}}$ [mol/(s m^2^)] is the total CO_2_ molar flux absorbed, $P_{{\mathrm{CO}}_{2}}^{\mathrm{gas}}$ [Pa] is the partial pressure of carbon dioxide in the bulk gas and $P_{{\mathrm{CO}}_{2}}^{\mathrm{liq}}$ [Pa] the partial pressure of carbon dioxide in the bulk liquid. This constant identifies both the effect of the rate of reaction and the mass transfer of the CO_2_ and it is usually reported in this kind of experimental work. Hence, also the value of the $K_{\mathrm{ov}}$ is reported with its uncertainty.

### 2 Experimental design, materials, and methods

#### 2.1 Wetted Wall Column (WWC) experimental apparatus

**Fig. 1:**
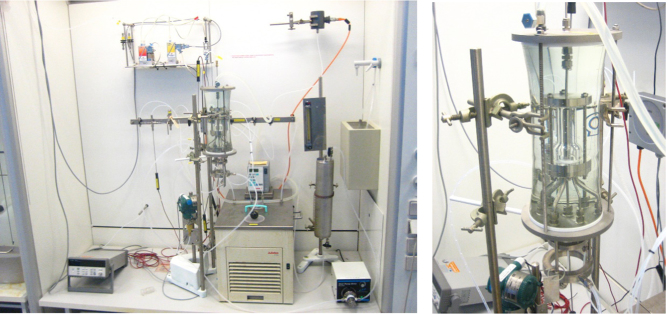
Wetted Wall Column set up pictures: (i) on the left the picture depicts the overall Wetted wall Column set up, (ii) on the right the picture shows a focus on the reaction chamber where the absorption reaction takes place.

Fig. 1 shows the experimental apparatus used in this work and Table 1 presents the instrument list. The Wetted Wall Column allows for counter-current contact between the liquid solvent and a defined gas mixture. CO_2_ and N_2_ are supplied by gas bottles with a molar purity of 99.995% and 99.996% respectively. The streams from the bottles are controlled by two Bronkhorst mass flow controllers which determine the composition of the inlet mixture in the chamber. The gas mixture passes through a pre-saturator at ambient temperature and a saturator immersed in a thermostatic bath at the temperature of the experiment. After the saturators, the gas mixture is saturated with water at the same temperature of the thermostatic bath. Between the reaction chamber and the CO_2_ concentration probe (VAISALA CARBOCAP GMT 221) is positioned a condenser. The saturators and the condenser are necessary in order to have a known amount of water in the gas and consequently determine accurately the amount of CO_2_ in the gas flow. Before the reaction chamber, in the gas line, there is a bypass valve. The valve allows to measure the carbon dioxide concentration before and after the absorption reactions switching the flow straight to the CO_2_ probe or to the reaction chamber.

**Table 1:** Instrumentation list.

**Quantity measured**	**Manufacturer**	**Model**	**Range**	**Uncertainty**
N_2_ Flow [Ndm^3^/min]	Bronkhorst	F-201 CV	0–20 [Ndm^3^/min]	0.005VR+0.001FS
CO_2_ Flow [Ndm^3^/min]	Bronkhorst	F-201 CV	0–2 [Ndm^3^/min]	0.005VR+0.001FS
Pressure [mbar]	Rosmount	2088	0–9900 [mbar]	0.001FS
CO_2_ concentration [dimensionless]	VAISALA	GMT 220	0–0.1 [dimensionless]	0.02VR+0.002
Temperature [°C]	TC Direct	Pt 100 1/3 DIN	− 50 °C to 200 °C	0.03VR+0.0005FS
Density [g/cm^3^]	Anton-Paar	DMA 4100	0–3 [g/cm^3^]	$5*10^{-5}[g/{\mathrm{cm}}^{3}]$
Viscosity [mPa s]	Anton-Paar	AMV 200	–	0.001[mPa s]

The thermostatic bath controls the temperature of the reaction chamber and the temperature of the inlet solvent in the reaction chamber. The solvent, prepared with a known NH_3_ concentration and CO_2_ loading is charged in a liquid reservoir of 0.7 dm^3^. A micro pump pushes the liquid with a controlled mass flow into the thermostatic bath and then into the reaction chamber. The reaction chamber consists of a glass tube in which a stainless steel tube is located (dimensions are reported in Fig. 1). The liquid, pushed inside the stainless steel tube, falls down in a thin film around the stainless steel tube. In this way the contact area between the liquid and the gas phase is well defined.

Based on the known contact area between the gas and the liquid, and the amount of CO_2_ absorbed in the chamber, it is possible to determine the dependence between the CO_2_ surface flux absorbed by the liquid and the CO_2_ partial pressure in the gas mixture at a fixed temperature. A clear scheme of the plant layout is available in the Fig. 1 in the second paragraph of the article which describes the experimental data analysis [1].

##### 2.2 Solvent preparation procedure

The solvent is identified by two numbers: (i) the ammonia concentration, defined as the initial weight of ammonia on the weight of water (wt. NH_3_/wt. H_2_O) and (ii) the CO_2_ loading defined as the ratio between the CO_2_ and the ammonia in the solution. The chemicals used to prepare the solutions are: deionized water, ammonia solution 28 wt% and ammonium bicarbonate NH_4_HCO_3_. The solvent is prepared gravimetrically with a balance with an accuracy of 0.01 g and a full scale of 1000 g. The ammonia concentration and the CO_2_ loading is calculated considering the apparent concentration of H_2_O, NH_3_ and CO_2_. Deionized water is considered pure H_2_O, ammonia solution is 28 wt% NH_3_ and 72 wt% H_2_O, and one molecule of NH_4_HCO_3_ is one molecule NH_3_ plus one molecule H_2_O plus one molecule CO_2_.

##### 2.3 Experimental procedure

The experimental procedure for measuring the overall mass transfer coefficient is described by the following points:

1. prepare the solvent with the desired ammonia concentration and loading;
2. measure the density and the viscosity of the solvent;
3. flush the system with nitrogen for at least 15 min, corresponding to more than 300 reaction chamber volumes;
4. introduce the solvent in the experimental setup and pump the solvent in the liquid circuit until it forms a stable and uniform liquid film on the cylinder in the reaction chamber;
5. set the temperature of the thermostatic bath and wait that the temperature of the reaction chamber is stable at the desired value;
6. when the CO_2_ probe reports a null concentration of CO_2_ the set-up is ready for the experiment;
7. send a flow of N_2_ and CO_2_ with the desired concentration into the bypass gas line and measure the CO_2_ concentration at steady state condition;
8. direct the gas to the reaction chamber and measure the CO_2_ concentration at steady state condition;
9. repeat the last two points for four other different gas mixtures;
10. measure five points at different concentration of CO_2_ following the procedure here described in the points 7–9;
11. flow nitrogen in the set-up for 20–30 min and at least 10 l of water in order to clean up the set-up;

This procedure has been repeated at temperatures of 15 °C, 25 °C and 35 °C, ammonia concentrations of 5%, 10% and 15% and CO_2_ loadings of 0.2, 0.4 and 0.6 (0.5 for 15% of ammonia to avoid salt precipitations) for a total of 27 cases (3 × 3 × 3).
