# Supplementary material for: Experimental data of the aqueous NH3 and CO2 absorption at temperatures from 15 °C to 35 °C, NH3 concentrations from 5% to 15% and CO2 loadings from 0.2 to 0.6 measured with the Wetted Wall Column
Source: Data Brief. 2018 Mar 2;17:1240–4. doi: 10.1016/j.dib.2018.02.047 (PMC6099518; doi:10.1016/j.dib.2018.02.047)
Supplement: Supplementary file 1 — Transparency document [file mmc1.docx]

Dear Editor,

authors of the manuscript do not have any potential conflicts of interest include employment, consultancies, stock ownership, honoraria, paid expert testimony, patent applications/registrations, and grants or other funding.

Best regards,

Davide Bonalumi
